# HFD-induced Alterations in Renal Tubular Oatp4c1-P-gp Transport Systems in Mice: Impact on Digoxin Renal Excretion and Gadolinium-Enhanced Radiological Manifestations

**DOI:** 10.2174/0113892002371501250610074757

**Published:** 2025-06-23

**Authors:** Jingwen Men, Jing Li, Tianyan Zhang, Yang Chen, Bin Xu, Huinan Hou, Lu Sun, Haoran Yue, Zhaoyue Duan, Ting Gui, Zhibo Gai

**Affiliations:** 1 Innovative Institute of Chinese Medicine and Pharmacy, Shandong University of Traditional Chinese Medicine, Jinan, 250355, China;; 2 Department of Pathology, Changqing District People's Hospital of Jinan, 250300, China;; 3 Department of Pharmacy, the Second Affiliated Hospital of Shandong First Medical University, Taian, 271000, China;; 4 School of Medicine, Shandong University of Traditional Chinese Medicine, Jinan, 250300, China;; 5 Experimental Center, Shandong University of Traditional Chinese Medicine, Jinan, 250300, China;; 6 Department of Clinical Pharmacology and Toxicology, University Hospital Zürich, University of Zürich, 8006, Zürich, Switzerland;; 7 Key Laboratory of Traditional Chinese Medicine Classical Theory, Ministry of Education, Shandong University of Traditional Chinese Medicine, Jinan, 250300, China

**Keywords:** Organic anion transporting polypeptide 4c1 (Oatp4c1, Slco4c1), P-glycoprotein (P-gp), digoxin, pharmacokinetic, gadolinium-ethoxybenzyl-diethylenetriamine pentaacetic acid (Gd-EOB-DTPA), oatp4c1 function

## Abstract

**Objective:**

The clearance of digoxin in obese patients with renal impairment is reduced, leading to elevated serum concentrations and increased risks of digoxin toxicity. However, the exact mechanism of such alterations in obese patients remains unclear. Previous studies have suggested that the organic anion transporting polypeptide 4c1 (Oatp4c1, Slco4c1) mediates the elimination of digoxin at the basal membrane of the proximal tubule (PT), indicating its potential role in the pharmacokinetic changes in obese patients. This study aims to investigate the effects of a high-fat diet HFD on digoxin pharmacokinetics and transporter expression in mouse models and further analyze its significance by detecting the expression of transporters in human renal tissue samples.

**Methods:**

First, HFD-induced obese mouse model was established. Mice were intraperitoneally injected with digoxin, and 24-hour urine samples and blood samples at five time points were collected. Pharmacokinetic evaluation was performed using liquid chromatography-tandem mass spectrometry. Renal pathological changes and the expression of digoxin transporters (Oatp4c1 and P-glycoprotein (P-gp)) were assessed using histological staining, Western blots (WB), as well as quantitative polymerase chain reaction (qPCR). Human renal pathologic alterations and expression of transporter proteins showed consistency with the results of animal experiments. To explore the potential use of gadolinium-ethoxybenzyl-diethylenetriamine-pentaacetic acid (Gd-EOB-DTPA) as a marker for Oatp4c1 function, drug interactions between digoxin and Gd-EOB-DTPA were assessed in mice.

**Results:**

HFD-induced obese mice showed significant increases in body weight, blood glucose, and triglyceride, along with elevated blood concentration of digoxin, increased areas under the curve, reduced renal clearance rate (CLr), and prolonged half-life (t1/2). Histological staining revealed proximal tubular epithelial cell detachment and slight fibrosis in the kidney of the HFD group, with decreased expression of villin, the protein marker for PT. Immunofluorescent staining and Western blots for digoxin transporters showed a significant reduction of Oatp4c1 and P-gp proteins, suggesting that the renal elimination of digoxin was affected by the reduced level of Oatp4c1 and P-gp proteins. Co-administration of digoxin and Gd-EOB-DTPA resulted in a reduced clearance of Gd-EOB-DTPA, suggesting that both share the same transporter. The blood concentration of Gd-EOB-DTPA was higher (77.5%) in the HFD group. Renal magnetic resonance imaging (MRI) intensity was lower in the HFD group after Gd-EOB-DTPA administration compared to the Chow group.

**Conclusion:**

Obesity-induced kidney damage results in decreased Oatp4c1 and P-gp expression and function in PT, resulting in a reduction of digoxin renal clearance. The inhibition of Gd-EOB-DTPA clearance by digoxin co-administration and the increased Gd-EOB-DTPA blood concentration in the HFD group both suggest its potential use in characterizing the Oatp4c1 function *in vivo*.

## 1 INTRODUCTION

The burgeoning global obesity epidemic and its associated complications, notably kidney disease, have become a critical focus in public health [1-3]. Obesity confers a significant risk for renal impairment, with the incidence of kidney disease in obese individuals ranging from 18-54%, and this rate nearly doubles when obesity is comorbid with metabolic syndrome [1, 4]. Given the pivotal role of kidneys in drug excretion, obesity-related renal dysfunction can profoundly impact the pharmacokinetics of medications that are primarily cleared by the kidneys, such as digoxin. In patients with impaired renal function, reduced renal clearance of digoxin leads to increased blood levels, heightened toxicity risk, and an extended half-life [5]. Despite the clinical implications, the mechanisms behind the altered renal clearance of digoxin in the context of renal impairment are not fully understood. Models of obesity-induced pharmacokinetic changes in drugs are lacking at this stage, so it is extremely important to understand the causes of pharmacokinetic changes.

Early insights into renal clearance of digoxin, as demonstrated by Koren *et al.* in 1987, challenged the traditional glomerular filtration-centric view, highlighting the significant role of tubular secretion in its elimination [6-7]. Firstly, despite accounting for the 25% protein binding of digoxin, its clearance surpasses inulin clearance by 46 to 94%, suggesting that digoxin is affected by tubular secretion [8]. Secondly, the Multiple Indicator Dilution Technique (MIDT) enables direct measurement of digoxin's transtubular transit [9]. Thirdly, several drugs co-administered with digoxin (*e.g.*, quinidine, verapamil, amiodarone, and cyclosporine) significantly reduce its renal clearance after GFR adjustment [10-15]. Moreover, in the micropuncture study, a reversible concentration gradient of digoxin is transported from the basolateral to the apical membrane [16-17]. All of the above evidence suggests that some of the digoxin is cleared by proximal tubular transport.

The organic anion-transporting polypeptide 4c1 (Oatp4c1, Slco4c1) and P-glycoprotein (P-gp, MDR1) are key players in digoxin transport, with Oatp4c1 facilitating digoxin entry into the renal proximal tubular lumen and P-gp mediating its efflux into urine [18-19]. While the role of P-gp is well-documented, the function of Oatp4c1 in the context of obesity-induced renal injury requires further exploration. Emerging data suggest that early kidney injury can affect the expression and function of drug transport proteins in the PT, potentially impacting the pharmacokinetics of digoxin [20-24]. This study hypothesizes that obesity-induced renal injury may disrupt Oatp4c1 function, leading to reduced renal clearance of digoxin. By employing a high-fat diet (HFD)-induced obese mouse model, we aim to delineate the pharmacokinetic changes associated with early renal injury due to obesity and to uncover the mechanisms behind the observed alterations in digoxin pharmacokinetics.

In addition, Gd-EOB-DTPA is mainly used clinically to diagnose altered liver function as a common substrate of the OATPs and the MRPs. MRI signaling of Gd-EOB-DTPA was also found to be expressed in the kidneys in both human and animal studies [25-28]. The DrugBank database revealed a drug interaction between Gd-EOB-DTPA and digoxin. However, this database does not provide detailed experimental data and results. Therefore, this study hypothesized that Gd-EOB-DTPA and digoxin may share the same transport protein in the kidney [21]. The idea of using Gd-EOB-DTPA as a characterization of Oatp4c1 visualization to observe changes in digoxin pharmacokinetics still requires further validation.

## 2 MATERIALS AND METHODS

### 2.1 Animals

Prior to experiments, six-week-old male C57BL/6 mice (20-22 g), purchased from Charles River Laboratories (Beijing, China), were acclimated for seven days in a special pathogen-free facility (SPF) with adaptive feeding. All mice were provided with a temperature of 25 ± 1 °C and a 12 h light-dark cycle, as well as prescribed food and water. First, mice were randomly assigned to either a normal diet (Chow) or a high-fat diet (HFD) group. The standard pelleted diet (TK1004, Beijing Ke-ao Xie-li Feed) was fed to the Chow mice, while the HFD mice were given a diet designed to induce obesity (NO47, Beijing Ke-ao Xie-li Feed). The high-fat diet provided 59.28% of its total energy from fat, with an energy density of 5.335 kcal/g.

### 2.2 Determination of Organ Index

According to the body mass (g) of mice and the total mass (mg) of bilateral kidneys, the kidney index is calculated using formula (1). The mass of fat (epididymal fat, perirenal fat, and brown fat) was measured, and the fat mass index was calculated according to formula (2).


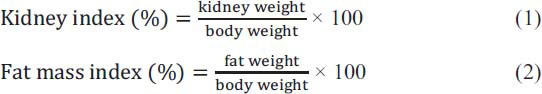


### 2.3 Pharmacokinetics Studies of Digoxin

Digoxin (CHG0575, Keli, Sanofi (Hangzhou) Pharmaceutical Co., Ltd., China) was administered intraperitoneally (0.05 mg/kg) to mice as a bolus dose. Doses even higher than these values have been shown to result in nontoxic serum concentrations [29]. After drug administration, the mice were placed into clean metabolic cages. A device for collecting urine was placed at the bottom of each metabolic cage. Urine samples within the time interval of 0 - 24 hours were collected. The collected urine samples were then transferred into centrifuge tubes and centrifuged at a speed of 1,000 revolutions per minute for 10 minutes in a centrifuge. Six mice per group were euthanized by lethal blood sampling. At each time point, blood samples were collected and allowed to clot while being kept on ice. After separating the plasma, they were centrifuged at 3500 rpm for 15 minutes and then analyzed. The levels of digoxin in serum (0 minute, 15 minutes, 45 minutes, 60 minutes, and 120 minutes) and urine (within 24 hours) were measured by liquid chromatography-mass spectrometry/mass spectrometry (LC-MS/MS) at Shandong Agricultural University. All pharmacokinetic parameters were calculated using professional pharmacokinetic software (DAS 2.0). Body clearance (CL/F, F=bioavailability) was calculated using the following formula: CL/F=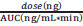, where F is a constant because the same formulation of digoxin (as a solution) was used, and it is assumed that bioavailability remains consistent across different mice.

### 2.4 Biochemical and ELISA Analyses

Serum total cholesterol (TC), serum creatinine (SCR), blood urea nitrogen (BUN), and urine protein (mAlb) were measured using cobas^®^ 4000 analyzer series (Roche, Basel, Switzerland).

### 2.5 Histological Examination

The kidneys were collected, bifurcated lengthwise, and preserved in 4% paraformaldehyde and embedded in paraffin. Human kidney tissue samples were provided by the Human Biomedical Research Ethics Committee of Shandong Provincial Hospital, Affiliated with Shandong University (NSFC: NO. 2020-154). Samples (n=6) embedded in paraffin were sectioned into 4 μm thick pieces, adhered to slides, and stained using hematoxylin and eosin (HE, EE0012, Spark Jade) for a comprehensive histological evaluation. The samples were examined under an inverted microscope-imaging system (CKX53+C2, Olympus) to determine the presence of glomerular and tubular changes.

### 2.6 Immunostaining

Kidney sections were cut at 4 μm, and paraffin sections were immunostained using a microwave-based antigen retrieval method. In this study (n=6), one polyclonal antibody was used, *i.e.*, villin (16488-1-AP, Proteintech) [30]. The samples were examined under an inverted microscope-imaging system (CKX53+C2, Olympus) to determine the presence of tubular injury.

### 2.7 Immunofluorescence

Antigen retrieval was the initial step in the preparation of mouse renal (4 μm thick) and human kidney tissue sections (4 μm thick), as mentioned above. The samples (n=6) were first microwaved in a 10 mM citrate buffer (EE0005, Spark Jade) at pH 7.4 for 30 minutes and then allowed to cool in PBS for two hours. The application of 3% H_2_O_2_ for 15 minutes, following three PBS washes, inhibited nonspecific protein binding. Following that, paraffin sections were incubated for an entire night at 4°C in a humidified chamber using the diluted primary antibodies, namely villin (1:200), Slco4c1 (1:300, 24584-1-AP, Proteintech), and P-gp (1:200, 22336-1-AP, Proteintech). After removing the primary antibodies, the sections were rinsed three times with PBS and then incubated with the Goat Anti-Rabbit IgG (H+L)-Alexa Fluor 488 (EF0008, Spark Jade) or Goat Anti-Rabbit IgG (H+L) - Alexa Fluor 594 (EF0011, Spark Jade) for one hour at room temperature in the dark. By mixing 4′,6-diamidino-2-phenylindole dihydrochloride (DAPI) (EE0011-B, Spark Jade), nuclei were stained. After that, the slides were covered with mounting medium (Dako, USA) and sealed with coverslips. Using a fluorescence microscope (Axio Imager. A 2, ZEISS), the positive signals of villin, Slco4c1, and P-gp were observed.

### 2.8 Western Blotting

Kidney tissues were homogenized in radioimmunoprecipitation assay (RIPA) buffer (EA0002, Spark Jade) containing protease and phosphatase inhibitors, then centrifuged at 13,500 × rpm for 15 minutes at 4°C. Following a 10-minute denature period at 95°C, 60 μg of clear kidney lysates were electrophoresed in polyacrylamide gels containing 10% SDS. Proteins were transferred to 0.45 μm PVDF membranes and blocked for 4 hours with 5% nonfat dry milk. The primary antibody was then left on the membranes for one night at 4°C, and the HRP-conjugated AffiniPure Goat Anti-Rabbit IgG (H+L) (SA00001-2, Proteintech) was left on the membranes for one hour at room temperature. Antibodies used in this study included the polyclonal antibody villin (16488-1-AP, Proteintech), the polyclonal antibody Slco4c1 (24584-1-AP, Proteintech), and the polyclonal antibody P-glycoprotein (22336-1 -AP, Proteintech).

### 2.9 qPCR

RNA from treated cells and tissues was extracted using TRIzol (Invitrogen, CA, USA) according to the manufacturer's instructions. Then, RT and qPCR were performed according to the previously described protocol. Briefly, reverse transcription of 2 μg of RNA was performed by treating with DNAse (Promega), primed with oligo-dT, and with Superscript II (Invitrogen). qPCR was performed by using TaqMan master mix (Applied Biosystems, CA, USA) and first-strand complementary DNA as a template. The Slco4c1 (Mm00554204_m1), p-gp (Mm00456156_m1), and villin (Mm00494146_m1) probes were purchased from Applied Biosystems (CA, USA). mRNA expression was reported using the 2^−ΔΔCT^ method in two independent experiments. Actin was used as the housekeeping gene.

### 2.10 Pharmacokinetic Analysis of Drug Interactions

In the Simplex Gd-EOB-DTPA group, after anesthesia, a tail vein catheter was placed in the mice, body temperature (33.5+/-0.5) and respiration (~30bpm) were monitored, and a Gd-EOB-DTPA contrast agent (HVJCH, Spark Jade) was selected and administered at a ratio of 0.025 mmol/kg at 1.0 mL/s contrast injection flow rate. For the co-administered group, the dosing regimen of Gd-EOB-DTPA was the same as above, with the addition of digoxin (0.05 mg/kg). Gd-EOB-DTPA levels in serum were determined by the LC-MS/MS at Shandong Academy of Agricultural Science at 4 time points: 0 minute, 3 minutes, 15 minutes, and 45 minutes. Six mice per group were euthanized by lethal blood sampling. At each time point, samples were collected and allowed to clot while being kept on ice. After separating the plasma, they were centrifuged at 3500 rpm for 10 minutes and then analyzed. All pharmacokinetic parameters were calculated using professional pharmacokinetic software (DAS 2.0).

### 2.11 *In Vivo* MRI General Protocol

Imaging was performed after mice were weighed and anesthetized placed for the insertion of a tail vein catheter. During the data collection process, the length of the catheter, *i.e*., 8 cm, enabled the injection of contrast agent, which consisted of polyethylene tubing (PE-10) and 30-gauge needles. Body temperature and respiration rate were performed (SA Instruments Inc. NY) and maintained at approximately 33°C and 30 breaths per minute, respectively. Images were acquired using a 9.4T Bruker Biospec 70/30 USR with a RAREst sequence (TE:4.66 ms, TR:200 ms, 82 mm). Gd-EOB-DTPA (0.025 mmol/kg) (SJ-MB0285, Spark Jade) was injected into two groups of mice, and MRI images were obtained at two time points, 0 and 10 minutes, after injection.

### 2.12 Ethical Statement

The animal protocol was approved by the Animal Ethics Committee of Shandong University of Traditional Chinese Medicine (protocol number: SDUTCM20210707002) in compliance with the Chinese Experimental Animals Administration Legislation’s provisions and general recommendations, as well as the US National Research Council’s Guide for the Care and Use of Laboratory Animals. This study also adhered to internationally accepted standards for animal research, following the 3Rs principle. The ARRIVE guidelines were employed for reporting experiments involving live animals, promoting ethical research practices. All procedures involving human subjects were approved by the Ethics Committee of Human Biomedical Research of Shandong Provincial Hospital Affiliated to Shandong University (NSFC: NO. 2020 - 154). Written informed consent was obtained from all participants before their inclusion in the study. All patient information was anonymized to protect privacy, and only coded samples were used in the study. During data analysis and result reporting, no information that could identify the patients was involved.

### 2.13 Statistical Analysis

Statistical analysis was performed using GraphPad Prism 9 software. All data were presented as a mean ± SD. Differences among groups were analyzed by a two-way ANOVA, followed by a post hoc test for significant differences. A *p*-value of < 0.05 was considered significant.

## 3 RESULTS

### 3.1 High-fat Diet Induced Weight Gain, Elevated Blood Glucose, and Abnormal TG in Obese Mice

**Fig. (1):**
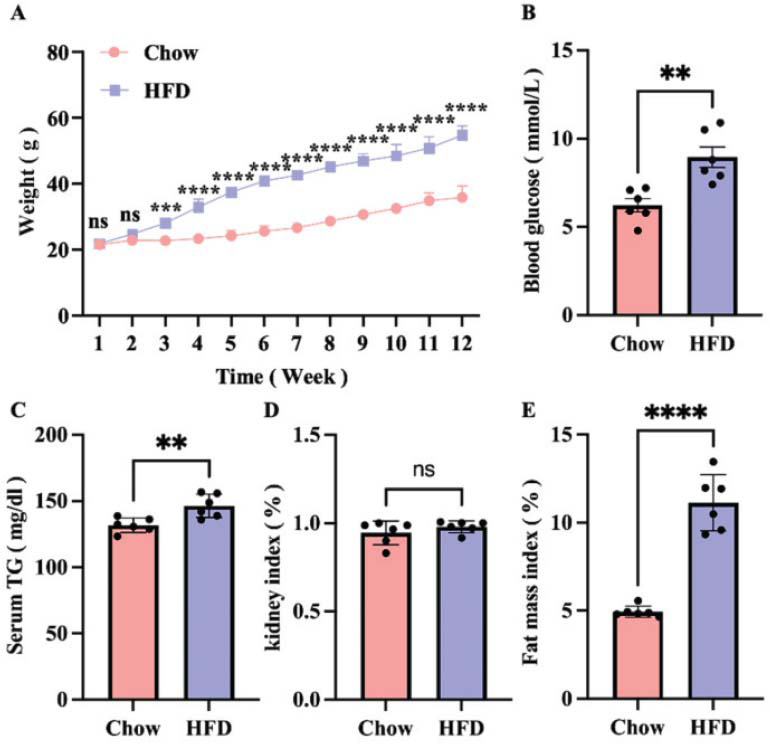
The obesity model was successfully established. The body weight (**A**), blood glucose levels (**B**), blood TG levels (**C**), kidney index (%) (**D**), and fat mass index (**E**) of different groups. Chow, normal standard diet; HFD, high-fat diet. Values are means ± SD (n = 6). ****p < 0.0001; **p < 0.01; ns: not significant.

To monitor the progression of HFD-induced obesity, body weight and fasting blood glucose were measured weekly. As shown in Fig. (**1A**), the HFD group exhibited accelerated weight gain after two weeks of feeding, which was significantly greater than the Chow group. After 16 weeks of feeding, the blood glucose (*p*<0.01), triglyceride (*p*<0.01) levels, and fat mass index (p<0.0001) in the HFD group were significantly higher than those of the Chow group (Figs. **1B**, **1C**, **1D**, and **1E**). However, there was no significant difference in the kidney index (Fig. **1D**). HE staining of the liver showed that lipid droplet accumulation occurred in the HFD group (Fig. **S1**). The above results all confirmed the obesity induced by HFD.

### 3.2 Digoxin Plasma Concentration Increased, Renal Clearance Decreased, and AUC Increased in the HFD Group

**Fig. (2):**
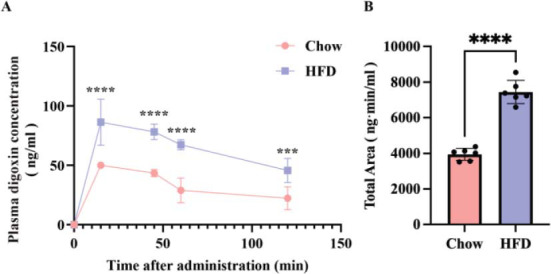
The difference in the blood concentration of digoxin between the Chow group and the HFD group of mice. (**A**). Digoxin (0.05 mg/kg) was administered intraperitoneally to HFD mice (solid squares) and normal mice (solid circles). Digoxin plasma concentrations were monitored using LC-MS/MS until 120 minutes after administration. (**B**) Significant differences in AUC between the two groups of mice. Values are means ± SD (n = 6). **** *p* < 0.0001; *** *p* < 0.001.

Both Chow and HFD received intraperitoneal administration of digoxin (0.05 mg·kg-1), and digoxin plasma concentrations were determined (Fig. **2A** and **B**). According to the results, the peak plasma concentration of digoxin in both groups was reached 15 minutes after administration and declined steadily thereafter. Table **1** lists the pharmacokinetic parameters. The pharmacokinetic changes in the HFD group were specifically manifested as a significant increase in AUC_(0-t)_, obvious decreases in CL/F and renal clearance, and a prolonged t_1/2_. In the Chow group, the AUC_(0-t)_ of digoxin decreased to approximately 35.3% from that in the HFD group (Table **1**). Meanwhile, the CL/F of Chow mice was about 2.5 times that of the HFD group. The CLr in the HFD group was reduced to 23.5 % of that in the Chow group, with a 1.6-fold prolongation of t_1/2_.

### 3.3 The Decrease in Digoxin Renal Clearance in the HFD Group was not Related to Glomerular Filtration

**Fig. (3):**
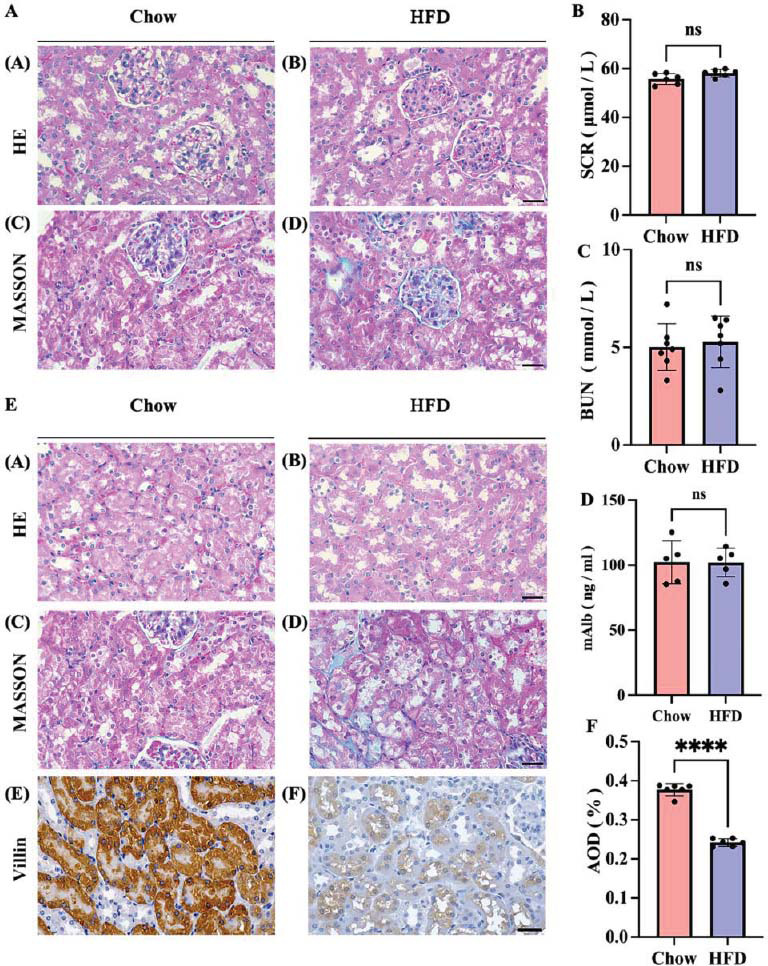
High-fat diet induced kidney injury in mice. (**A**) Histological analysis of glomeruli in Chow group (a and c) and HFD group (b and d) by HE staining (a and b) and Masson's Trichrome staining (c and d). (**B**) Serum levels of creatinine. (**C**) Blood urea nitrogen (BUN). (**D**) Urine protein (mAlb). (**E**) Histological analysis of PT in Chow group (a, c, and e) and (**F**) HFD group (b, d, and f) by HE staining (a and b), Masson's Trichrome staining (c and d), and immunostaining for villin (e and f). Black bars represent 50μm. F. The AOD (%) of villin in the Chow group and HFD group was analyzed. Values are means ± SD (n = 6). **** *p* < 0.0001; ns. not significant.

Given that digoxin is a drug primarily excreted through glomerular filtration and renal tubular secretion, the examination of renal structure is essential for exploring the aforementioned changes. To examine if digoxin elimination is affected by glomerular filtration, glomerular structures were first analyzed. Under HE staining, the glomeruli appeared pink in color and contained vascular lumens, endothelial cells, basement membranes, and the outer layer of mesangial cells without fibrosis and inflammatory cell infiltration (Fig. **3A**). Serum creatinine, blood urea nitrogen (BUN), and urine protein (mAlb) also indicated no significant differences (Figs. **3B**-**D**). HE staining, Masson's Trichrome staining, and blood creatinine levels indicated that the changes in digoxin blood levels were not due to glomerular filtration, which is consistent with other studies [31].

### 3.4 Following Renal Tubule Injury, Reduced Expression of the Oatp4c1-Pgp Transmembrane Transport System was Observed, which Affected the Digoxin Renal Clearance

In the early stages of renal injury, changes in the structure of PT (Proximal Tubule) and the related basolateral or apical membrane proteins lead to a downregulation in the expression and function of various drug transporter proteins (for example, Slc34a1, Slc34a3, Slc20a2, Slc5a2, Slc5a3, Slc5a8, Slc6a20, Slc27a2, Slc12a3, slc38a2, *etc*.) [32-34]. To elucidate if changes in PT are the cause of altered digoxin elimination, we focused on the structures of PT. In the Chow group, tubules were tightly arranged with intact basement membranes, and no fibrosis or inflammatory cell infiltration was observed (Fig. **3E**). In the HFD group, PT showed signs of damage, including irregular lumen shapes and epithelial cell shedding (Fig. **3E**). Significant interstitial collagen fiber deposition was observed in the kidneys of the HFD group *via* Masson staining (Fig. **3E**). Villin was used as a marker for PT. Immunostaining for villin revealed a reduced villin-positive signal in the HFD group *versus* the Chow group (Fig. **3F**). In contrast, the kidneys of the Chow group exhibited clear tubular, well-arranged tubular endothelial cells and no signs of fibrosis or inflammatory cell infiltration *via* HE staining. Thus, in early obesity, PT damage in the kidney precedes glomerular injury [23, 35]. HE staining, Masson’s Trichrome staining, and villin staining indicated early tubular damage in obese mice.

**Fig. (4):**
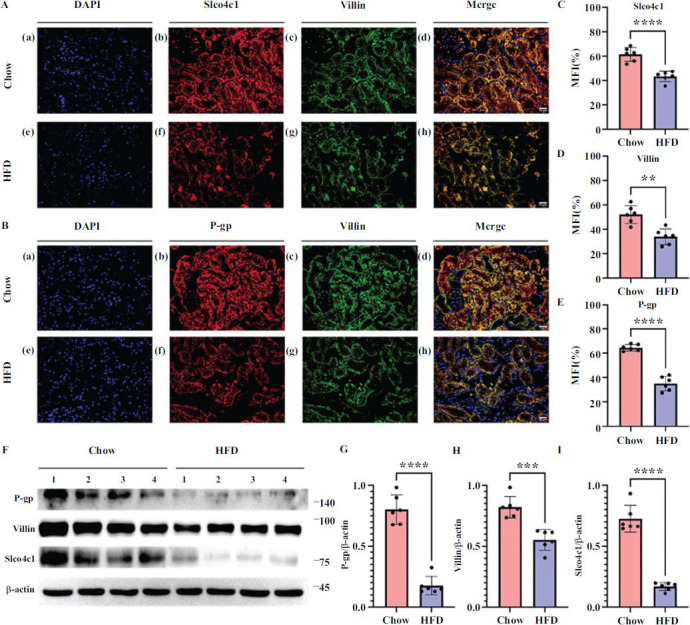
Expression levels of villin, Slco4c1, and P-gp were down-regulated in the renal cortex of the HFD group. (**A**) Kidney slices from mice were stained with DAPI (a, e), Slco4c1 (b, f), and villin (c, g). Merged images show co-localization of DAPI/Slco4c1/villin (d, h). The DAPI signal is shown in blue, the villin signal is shown in green, and the Slco4c1 expression in red. White bars represent 50μm. (**B**) Kidney slices from mice were costained with DAPI (a, e), P-gp (b, f), and villin (c, g). Merged images show co-localization of DAPI/P-gp/Villin (d, h). The DAPI signal is shown in blue, the villin signal is shown in green, and the P-gp expression in red. White bars represent 50μm. C-E. The average fluorescence intensity of Slco4c1 (**C**), villin (**D**), and P-gp (**E**) in the Chow group and the HFD group was statistically analyzed. (**F**) Representative images of kidneys by Western blot for P-gp, villin, Slco4c1, and actin from the Chow group (left) and the HFD group (right). G-I. Analysis of relative ratio for P-gp/actin (**G**), villin/actin (**H**), and Slco4c1/actin (**I**). Values are mean ± SD (n = 6). **** *p* < 0.0001; *** *p* < 0.001; ** *p* < 0.01.

Therefore, the decrease in the renal clearance rate of digoxin in mice may be related to the decrease in the expression of Slco4c1 and P-gp. To explore this, we examined the locations of Slco4c1 and P-gp by immunofluorescent (IF) staining. The results showed that Slco4c1 was located on the basolateral, while P-gp was located on the apical side of PT (Figs. **4A** and **4B**). Compared to the Chow group, the HFD group showed reduced intensity of Slco4c1 (*p*<0.0001), villin *p*<0.01), and P-gp (*p*<0.0001) in the PT, indicating a reduction of Oatp4c1-P-gp protein levels in the kidneys of HFD-induced obese mice (Figs. **4C**, **4D** and **4E**). Subsequently, WB and PCR were used to confirm the reduced expression levels of Slco4c1 (*p*<0.0001), P-gp (*p*<0.001), and villin (*p*<0.0001) in the kidneys of HFD mice (Figs. **4F**, **4G**, **4H**, **4I** and Fig. **S2**). These results collectively suggest that HFD-induced early injury to the PT in obese mice led to decreased expression of Oatp4c1 and P-gp, which, in turn, may affect the renal clearance of digoxin.

### 3.5 Common Features of Pathological Changes in Human and Mouse Kidneys

**Fig. (5):**
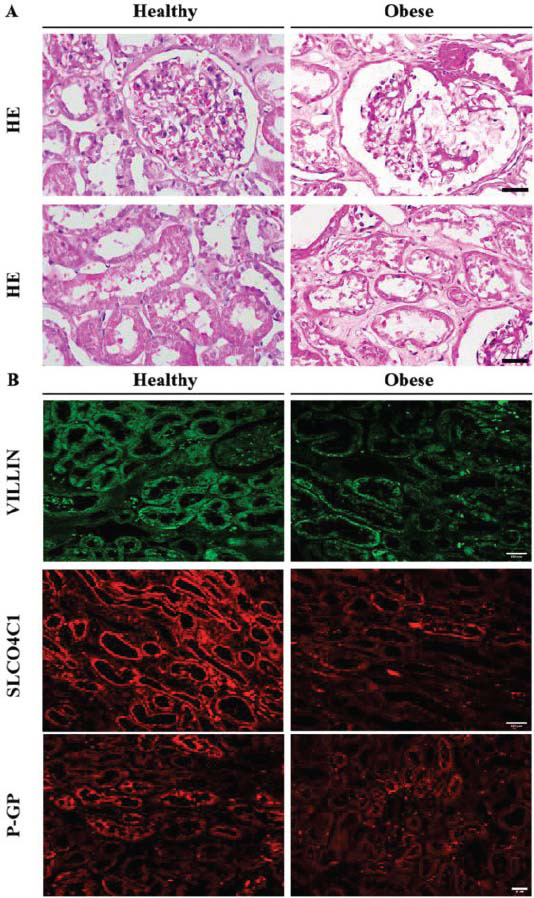
The changes in OATP4C1-P-GP in obese patients. (**A**) HE staining of glomeruli and renal tubules in healthy and obese patients. Black bars represent 50μm. (**B**) Kidney slices from healthy and obese patients were stained with villin, along with Slco4c1 and P-gp. White bars represent 20μm.

In this study, human kidney samples were used to observe pathological changes in renal tissues as well as renal tubular Oatp4c1 and P-gp expressions. In the obese group, the glomeruli showed only mild nonspecific changes, but the renal tubules showed tubular injury (Fig. **5A**). In (Fig. **5B**), it can be observed that fluorescence staining of normal renal tissues showed strong positive signals for villin, Slco4c1, and P-gp. Compared with the control group, the signal intensity of villin, Slco4c1, and P-gp was reduced in the obese group. The above results were consistent with the results of animal experiments. This suggests that human PT injury also affects villin, Slco4c1, and P-gp protein expressions.

### 3.6 Digoxin-Gd-EOB-DTPA Interaction and Decreased Clearance of Gd-EOB-DTPA in the HFD Group

**Fig. (6):**
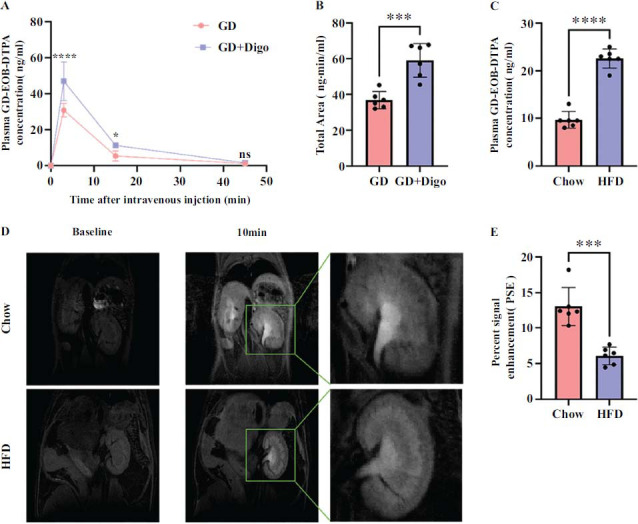
Plasma levels of Gd-EOB-DTPA and images of contrast-enhanced MRI. (**A**) Plasma levels of Gd-EOB-DTPA in mice intravenously co-injected with/without digoxin. (**B**) The AUC of plasma Gd-EOB-DTPA in mice intravenously co-injected with/without digoxin. (**C**) Plasma Gd-EOB-DTPA levels between the Chow group and the HFD group after 15 minutes of intravenous Gd-EOB-DTPA injection. (**D**) MRI of Chow and HFD group before and after the injection of Gd-EOB-DTPA. Each image consists of a baseline image (left), an image taken 10 minutes later after injection (t = 10; middle), and an enlarged image (right). (**E**) PSE of the MRI signals in Chow group and HFD group. Values are means ± SD (n = 6). **** *p* < 0.0001; *** *p* < 0.001; ** *p* < 0.01; ** *p* < 0.05; ns. not significant.

In this study, we examined the pharmacokinetic profile of Gd-EOB-DTPA in the combination group (Gd-EOB-DTPA + digoxin) (Fig. **6A** and Fig. **6B**). Table **2** summarizes the pharmacokinetic parameters of the two groups. Fig. **6** shows that the clearance of Gd-EOB-DTPA was reduced by 72.3% in the co-administered digoxin group compared to the tail vein Gd-EOB-DTPA group alone. The clearance of Gd-EOB-DTPA was decreased in the co-administration group. This may be due to the fact that both digoxin and Gd-EOB-DTPA were transported *via* Oatp4c1, and Gd-EOB-DTPA was inhibited by digoxin.

In this study, blood concentrations of Gd-EOB-DTPA were higher in the HFD group than in the Chow group (Fig. **6C**). To monitor the direct effect of renal uptake of Gd-EOB-DTPA *in vivo*, MRI was performed in the Chow and HFD groups of mice. Fig. (**6D**) shows the *in vivo* MRI results of kidneys in the Chow and HFD groups after injection of Gd-EOBDTPA. Before the drug injection, which was administered at time t=0, the kidney appeared dark in the baseline images of both groups of mice (Fig. **6D**). After 10 minutes of injection, the difference in kidney signals between the two groups became clear. The percentage of signal enhancement (PSE) is characterized by the difference between the two groups 10 minutes after Gd-EOB-DTPA injection (Fig. **6E**). The intrarenal signal intensity in the kidneys of Chow mice was significantly stronger than in the kidneys of mice in the HFD group. The above results suggest that the presence of renal pathologic changes in the HFD group decreased the renal clearance of Gd-EOB-DTPA.

## 4 DISCUSSION

In an animal study, it was found that the clearance rate of digoxin gradually decreased with increasing age in mice [36]. Clinical research indicates that impaired renal function can prolong the half-life of digoxin in humans, increasing the risk of accumulation and toxicity [37-38]. Another clinical study pointed out that in the elderly, body weight is a key factor affecting the clearance rate of digoxin [39]. However, none of the above studies have explained the underlying mechanism. Digoxin is a substrate of hepatic organic anion transporting polypeptides (namely Oatp1b1, Oatp1b3, and Oatp1a2), most of which are demonstrated to be down-regulated in obese and/or NAFLD models by many studies including our group [40-42]. It was found that the increase in blood digoxin levels in obese mice was possibly due to the decreased OATPs. However, drug-drug interactions (DDIs) resulting in alterations in digoxin plasma levels have been largely attributed to changes in P-gp efflux activity in the gut, liver, and kidney [43-45]. Notably, hepatic P-gp induction during diet-induced obesity and/or the progression of NAFLD has been reported in several animal models, including the methionine- and choline-deficient diet and atherogenic diet models [46, 47], as well as in patients with NAFLD [48], which is consistent with our results (data not shown). The increased hepatic P-gp level, in turn, leads to faster biliary digoxin excretion [49, 50]. Overall, above mentioned changes in hepatic transporters cannot always predict the increase in blood digoxin levels in more complex systems, such as in obese animal models or even in freshly isolated primary hepatocytes.

This study offers novel insights into digoxin renal clearance, identifying the critical role of the renal basolateral membrane's Oatp4c1 transporter in modulating pharmacokinetics beyond glomerular filtration. Additionally, we observed alterations in renal transport proteins in mouse renal tissue due to a high-fat diet. To investigate the relevance and potential clinical implications of this finding in humans, we broadened our research scope to include clinical samples. The results indicated that a comparable trend of changes in transport proteins was also evident in human renal tissues, particularly among obese patients. Our findings revealed the influence of proximal tubular transmembrane transport on digoxin's reduced renal clearance, a factor not accounted for by traditional renal function metrics. Moreover, leveraging Gd-EOB-DTPA as an Oatp4c1 substrate, we present a novel biomarker strategy for pharmacokinetic monitoring, addressing the current absence of direct diagnostic tools for drug safety and dosage optimization.

Our research contributes to the understanding of digoxin renal clearance by elucidating that pharmacokinetic alterations are not solely due to age-related declines or impaired glomerular filtration, as previously indicated [36, 38, 51-52]. Despite digoxin's significant renal excretion rate of 60 to 80%, our observations align with recent studies suggesting that its pharmacokinetics are influenced by factors beyond glomerular filtration [53-56]. This implicates the transmembrane transport function of the PT as a critical, yet underexplored, component in digoxin's renal clearance.

This study provides critical insights into the pharmacokinetics of digoxin, particularly in obese mice, by demonstrating the impact of the Oatp4c1-P-gp transport system on its renal clearance. We observed a significant downregulation of P-gp and Oatp4c1 in renal histopathological changes, which preceded glomerular lesions in the HFD group, suggesting their key role in digoxin's reduced excretion. This finding is consistent with the established competition between P-gp substrates and digoxin for apical binding sites, reducing digoxin renal clearance [6]. However, our research extends beyond by highlighting the contribution of the basolateral membrane, an area previously unexplored.

During renal injury, the expression of transporters, such as BCRP, MRP2, MRP4, P-gp, OAT1, OAT3, OCT1, OCT2, OCT3, and Oatp4c1, is altered, affecting substrate excretion across the renal tubular membrane [57-61]. The collaborative function of Oatp4c1 and P-gp in facilitating substrate transport into the lumen is essential for the excretion of uremic toxins like GSA, ADMA, and trans-aconitate [62]. The upregulation of this system is linked to enhanced clearance capacity and reduced renal inflammation [63]. Conversely, uremic toxins can inhibit Oatp4c1 expression, affecting the excretion of uremic toxins in Chronic Kidney Disease (CKD) [64].

The expression of P-gp in the kidney exhibits variability. It was found to be downregulated in CRF rat models but no significant difference was observed in STZ-induced diabetic mice compared to normal mice [31]. This study confirmed the downregulation of the Oatp4c1-P-gp system in obese mice, paralleling findings observed in the CRF rat model [65]. The observed downregulation potentially contributed to the accumulation of uremic toxins and digoxin, elucidating the altered pharmacokinetics of digoxin in CKD.

According to the FDA, Gd-EOB-DTPA is a drug eliminated through both renal (50%) and hepatobiliary (50%) pathways. It has been demonstrated good safety in a Phase I clinical trial, with complete clearance within 24 hours even at high doses [26]. Currently, Gd-EOB-DTPA is primarily used in clinical diagnosis for identifying changes in liver function [66-68]. In both clinical trials and animal studies, Gd-EOB-DTPA has been identified as a common substrate for the OATP and MRP transporters, and its MRI signal can also be detected in the kidney, serving as a diagnostic indicator of renal fibrosis. Shuboni-Mulligan *et al.* [69] detected reduced OATP levels in a diabetic animal model using MRI and proposed that Gd-EOB-DTPA could serve as a non-invasive indirect evidence to predict changes in OATPs expression in the liver [69]. The OATP isoforms expressed in rodent kidneys include Oatp1a1, Oatp1b3 and Oatp4c1; however, the knockout of Oatp1a1 and Oatp1b3 did not affect the renal Gd-EOB-DTPA imaging enhancement in mice, suggesting that Oatp4c1 might be the key transporter for renal uptake of Gd-EOB-DTPA. Data from the current study indicated that the co-administration of digoxin reduced the clearance of Gd-EOB-DTPA, suggesting a potential competitive drug-drug interaction for the Oatp4c1-mediated renal tubular elimination. Compared to the Chow group, the plasma concentration of Gd-EOB-DTPA was significantly higher in HFD mice 15 minutes after injection, indicating reduced clearance, which was consistent with the observations in ob/ob mice 5 minutes after Gd-EOB-DTPA injection [69]. Additionally, imaging data showed reduced distribution of Gd-EOB-DTPA in HFD mice, likely due to decreased expression of Oatp4c1, which resulted in a reduction of Gd-EOB-DTPA transmembrane uptake. Therefore, this study suggests that MRI signal intensity of renal Gd-EOB-DTPA in the HFD model can provide experimental support for changes in Oatp4c1-P-gp transmembrane system-mediated digoxin elimination.

## STUDY LIMITATIONS

This study has several important limitations that must be acknowledged. While our findings suggest that Oatp4c1 may be involved in Gd-EOB-DTPA transport, based on indirect evidence including pharmacokinetic changes (increased blood concentration, prolonged half-life, reduced clearance) and MRI signal alterations that align with Oatp4c1 downregulation in HFD groups, we currently lack direct experimental evidence of this transport relationship. Crucially, we have not yet confirmed the specific binding and transport interaction between Gd-EOB-DTPA and Oatp4c1 through either functional *in vitro* assays or genetic knockout models. However, we are actively pursuing these validations through two key approaches: (1) establishment of an Oatp4c1-overexpressing HEK293 cell model, and (2) development of a kidney-specific Oatp4c1 knockout animal model. Addressing these limitations in future research will be essential to strengthen our findings and provide more definitive insights into the role of Oatp4c1 in Gd-EOB- DTPA transport and metabolism.

## CONCLUSION

Our research unveils the Oatp4c1-P-gp transport system as a key determinant in digoxin renal excretion, particularly in obese patients, offering an unprecedented therapeutic target and monitoring strategy. The innovative application of Gd-EOB-DTPA as a non-invasive biomarker for renal transporter activity heralds a new era in real-time pharmacokinetic monitoring, with the potential to significantly improve drug safety. While our study significantly advances the field, it also paves the way for future investigations, including the impact of Oatp4c1 gene modulation on drug transport rates, which is currently underway in our laboratory. These endeavors will further refine personalized medicine approaches and optimize clinical outcomes, ensuring enhanced patient care and safety.

## Figures and Tables

**Table 1:** The Pharmacokinetic Parameters of Digoxin (n=6).

**Group**	**Chow Group**	**HFD Group**	***p* Value**
Cmax (ng/mL)	53	118.5	< 0.0001
Tmax (min)	15	15	-
t_1/2_ (min)	43.175	69.315	< 0.001
kel (min^−1^)	0.016	0.01	< 0.0001
CL/F (L/min/kg)	7.504	2.995	< 0.001
AUC (0-t) (ng/mL*min)	5935.359	16807.309	< 0.0001
CLr (μL/min)	0.51	0.12	< 0.0001

**Table 2:** The Pharmacokinetic Parameters of Gd-EOB-DTPA (n=6).

**Group**	**Gd-EOB-DTPA**	**Gd-EOB-DTPA+Digoxin**	***p* Value**
Cmax (ng/mL)	35.5	27.5	< 0.001
Tmax (min)	3	3	
t_1/2_ (min)	6.3	8.278	< 0.0001
kel (min−1)	0.11	0.084	< 0.01
CL (L/min/kg)	24.238	22.223	< 0.05
AUC(0-t) (ug/mL*min)	612.163	693.826	< 0.01

## Data Availability

The data and supportive information are available within the article.

## LIST OF ABBREVIATIONS

BUN: Blood Urea Nitrogen
CKD: Chronic Kidney Disease
CLr: Clearance Rate
HFD: High-Fat Diet
PT: Proximal Tubule
qPCR: Quantitative Polymerase Chain Reaction
MRI: Magnetic Resonance Imaging
MIDT: Multiple Indicator Dilution Technique
TC: Total Cholesterol
SCR: Serum Creatinine
WB: Western Blots
RIPA: Radioimmunoprecipitation Assay
