# Supplementary material for: HFD-induced Alterations in Renal Tubular Oatp4c1-P-gp Transport Systems in Mice: Impact on Digoxin Renal Excretion and Gadolinium-Enhanced Radiological Manifestations
Source: Curr Drug Metab. 2025 Jun 23;26(2):136–48. doi: 10.2174/0113892002371501250610074757 (PMC12824865; doi:10.2174/0113892002371501250610074757)
Supplement: Supplementary file 1 [file CDM-26-2-136_SD1.pdf]

Supplementary Material

HFD-induced Alterations in Renal Tubular Oatp4c1-P-gp Transport Systems in Mice: Impact on Digoxin Renal Excretion and Gadolinium-Enhanced Radiological Manifestations

Jingwen Men[1,#], Jing Li[2,#], Tianyan Zhang[1,3], Yang Chen[1], Bin Xu[1], Huinan Hou[1], Lu Sun[1], Haoran Yue[1], Zhaoyue Duan[5], Ting Gui[1,*] and Zhibo Gai[4,6,7,*]

[1]Innovative Institute of Chinese Medicine and Pharmacy, Shandong University of Traditional Chinese Medicine, Jinan, 250355, China; [2]Department of Pathology, Changqing District People's Hospital of Jinan, 250300, China; [3]Department of Pharmacy, the Second Affiliated Hospital of Shandong First Medical University, Taian, 271000, China; [4]Experimental Center, Shandong University of Traditional Chinese Medicine, Jinan, 250300, China; [5]School of Medicine, Shandong University of Traditional Chinese Medicine, Jinan, 250300, China; [6]Department of Clinical Pharmacology and Toxicology, University Hospital Zürich, University of Zürich, 8006, Zürich, Switzerland; [7]Key Laboratory of Traditional Chinese Medicine Classical Theory, Ministry of Education, Shandong University of Traditional Chinese Medicine, Jinan, 250300, China

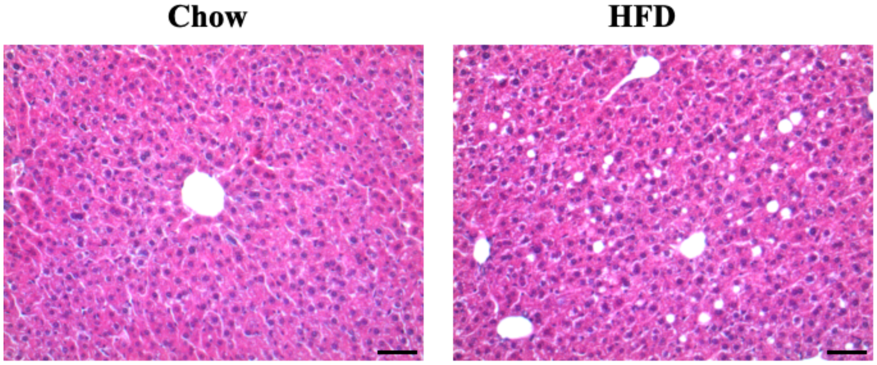

Fig. (S1). HE staining of the liver in the Chow group (left) and the HFD group (right). Black bars represent 50μm.

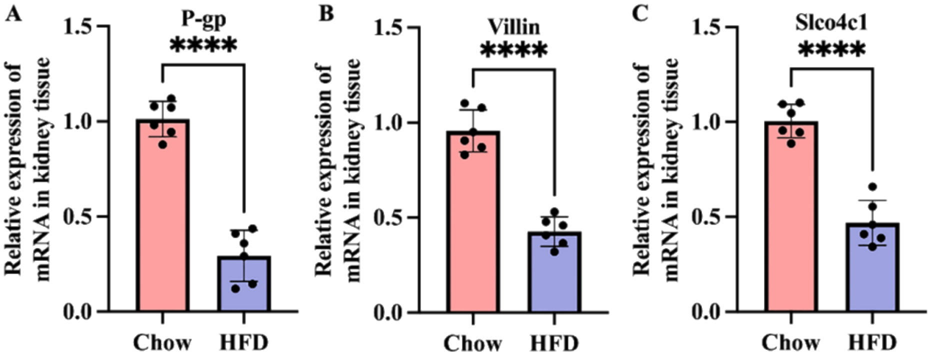

Fig. (S2). Differences in the expression of renal genes, P-gp (**A**), villin (**B**), and Slco4c1 (**C**), between the Chow group and the HFD group. Values are represented as means ± SD (n = 6). **** $p < 0.0001$.
